# Bubbled RNA‐Based Cargo for Boosting RNA Interference

**DOI:** 10.1002/advs.201600523

**Published:** 2017-03-27

**Authors:** Hyejin Kim, Jaepil Jeong, Dajeong Kim, Gijung Kwak, Sun Hwa Kim, Jong Bum Lee

**Affiliations:** ^1^ Department of Chemical Engineering University of Seoul 163 Seoulsiripdaero Dongdaemun‐gu Seoul 02504 Republic of Korea; ^2^ KU‐KIST Graduate School of Converging Science and Technology Korea University 145 Anam‐ro Seongbuk‐gu, Seoul 02841 Republic of Korea; ^3^ Center for Theragnosis Biomedical Research Institute Korea Institute of Science and Technology (KIST) Seoul 02792 Republic of Korea

**Keywords:** enzymatic synthesis, RNAi therapeutics, RNA nanoparticles, rolling circle transcription

## Abstract

As ribonucleic acid (RNA) nanotechnology has advanced, it has been applied widely in RNA‐based therapeutics. Among the range of approaches, enzymatically synthesized RNA structures for inducing RNA interference in cancer cells have potential for silencing genes in a target‐specific manner. On the other hand, the efficiency of gene silencing needs to be improved to utilize the RNA‐based system for RNAi therapeutics. This paper introduces a new approach for efficient generation of siRNA from bubbled RNA‐based cargo (BRC). The presence of bubbles in between to avoid nonfunctional short dsRNAs allows the RNA‐based cargoes to contain multiple Dicer‐cleavage sites to release the functional siRNAs when introduced to cells. BRCs can be synthesized easily in a one‐pot process and be purified by simple centrifugation. Furthermore, efficient target gene silencing by the bubbled structure is confirmed both in vitro and in vivo. Therefore, this bubbled RNA cargo system can be utilized for target‐specific RNAi therapeutics with high efficiency in the generation of functional siRNAs in the target cells.

Ribonucleic acid (RNA) has attracted considerable attention for its potential applications in nanomedicine for the treatment of cancer or infectious disease as well as for immunization.^1^ In addition to its range of inherent biological activities, molecular simplicity, and ease of modification have also accelerated the progress of RNA nanotechnology.^2^ In the early ages of nucleic acid engineering, most manipulating approaches aimed to generate DNA‐based materials.^3^ In comparison, RNA nanotechnology has progressed slowly due to the relatively high cost, existence of tertiary complex structures, and the susceptibility to nucleases.^4^ Nevertheless, RNA nanotechnology has continuously followed the trail of DNA nanotechnology to take advantage of the versatility of RNA.^5^ In particular, a number of synthetic approaches of RNA nanostructures have been reported for intracellular delivery of RNAi inducers.^6^

Rolling circle transcription (RCT)‐based enzymatic self‐assembly,^7^ which was recently adapted widely in the field of RNA nanotechnology, is one of the promising approaches for synthesizing RNA‐based materials. Since the introduction of the self‐assembly of RNAi‐inducing microsponge,^8^ the authors have previously reported a range of functional RNA structures, ranging from the nanometer‐scale to the millimeter‐scale, based on an enzymatic approach with the appropriate modifications; messenger RNA nanoparticles (mRNA‐NPs),^9^ RNA membrane,^10^ siRNA nanosheets,^11^ and tumor‐targeting RNA nanovector.^12^ Each structure was assigned with a distinct biological function, and each has shown its potential to be utilized in controlling the level of gene expression.

Among the abovementioned RNA structures, the efficiency of Dicer cleavage plays a critical role in inducing RNA interference by RNA‐based materials. Although the Dicer‐mediated generation of siRNA from a RNAi microsponge was ≈21% in a previous study,^8^ the formulation of the nanostructure was modified recently to 2D sheets to provide the enzyme with a wide surface area.^11^ This approach improved the efficiency of Dicer cleavage significantly to more than 80%. On the other hand, siRNA nanosheets have an irregular shape and a relatively wide size distribution owing to their uncontrollable preparation process involving ultrasonication. Here, a bubble generating complementary rolling circle transcription approach is introduced to induce the self‐assembly of bubbled RNA‐based cargoes (BRCs). The BRCs were designed rationally to bear multiple binding sites for the Dicer enzyme, while also having bubbles in between to prevent the Dicer from generating nonactive double‐stranded RNAs (dsRNAs). Moreover, the size of the BRCs is controlled by changing the ratio of the molar concentration of circular DNA to the unit concentration of T7 RNA polymerase. In addition, the resulting BRCs have a narrow size distribution with a polydispersity index (PDI) of ≈0.14, which indicates that the synthetic process is well‐conditioned and the BRCs are synthesized uniformly. The BRCs showed successful in vitro siRNA generation when introduced to Dicer and gene knockdown both in vitro and in vivo.

To synthesize BRCs, two types of closed circular DNAs were prepared, as reported previously.^13^ As illustrated in **Figure** **1**A, each type of circular DNA was preprogrammed to bear complementary DNA sequences to the following RNA sequences in the following order: (i) promoter for T7 RNA polymerase, (ii) sense or antisense strand for siRNA, (iii) nonhybridizing (bubble) region, and (iv) sense or antisense strand for siRNA (Table S1, Supporting Information). Both types of circular DNAs were introduced to the reaction mixture for RCT with T7 RNA polymerase and ribonucleotide triphosphates, and the sense and antisense strands for siRNA could be generated continuously by T7 RNA polymerase. As a consequence, the RNA strands transcribed from circular DNA1 can be hybridized partially with the RNA strand transcribed from circular DNA2, forming multiple Dicer‐cleavage sites (double stranded region) and bubble region in between. Multiple RNA strands then entangle with the neighboring strands and finally self‐assemble into nanosized sponge‐like particles.^8^

**Figure 1:**
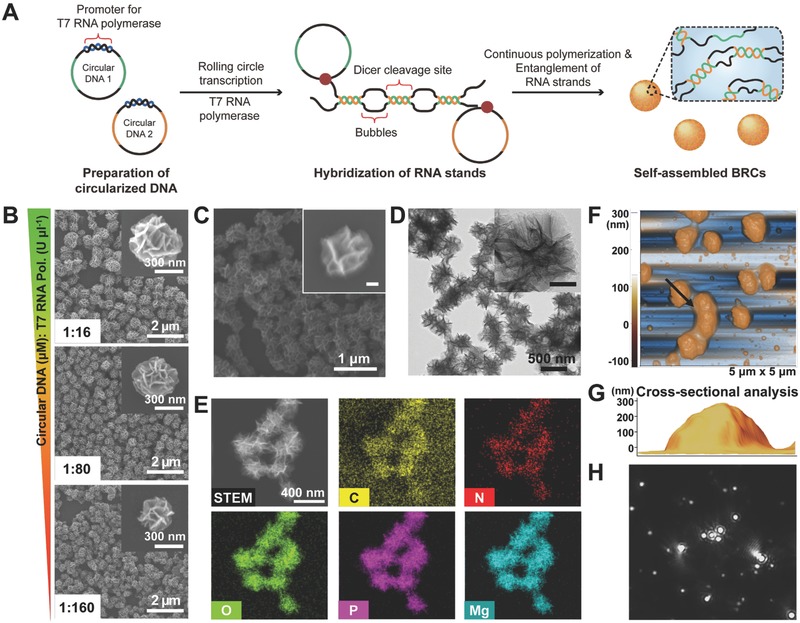
Schematic illustration of the formation process and characterization of BRCs. A) Primer DNA (blue)‐hybridized circular template DNAs were prepared. The sense and antisense strands for siRNA can be generated continuously from circular DNA1 and circular DNA2, respectively, by rolling circle transcription. The RNA strands can be hybridized to generate the double stranded region for the Dicer‐binding site and single‐stranded region (bubble region) in between. The RNA strands further entangle with the neighboring strands, finally resulting in the self‐assembled BRCs. B) SEM images of BRCs synthesized from different ratio of molar concentration of circular DNA to the unit concentration of T7 RNA polymerase in RCT reaction, ranging from 1:16 to 1:160. C) SEM and D) TEM images of BRCs. The inset scale bar indicates 100 nm. E) TEM‐based EDX analysis, revealing BRCs are made up of C, N, O, P, and Mg. AFM image shown in 3D, F) indicating overall height distribution of BRCs, and the cross‐sectional 3D image G) showing that the height of the nanoparticle is ≈350 nm. H) Captured image from Video S1 (Supporting Information), showing Brownian motion of the well‐dispersed nanoparticles.

One of the advantages of the synthetic approach of RCT‐induced self‐assembly is that the sizes and sequence‐dependent biological functions of the resulting materials can be manipulated easily. As observed in previous research,^14^ the size of the nanoparticles was controlled rationally by adjusting the enzyme concentration. The concentration of enzyme involved in the RCT reaction is not the only size‐determining factor, and the ratio of molar concentration of template circular DNA to the unit concentration of enzyme is a more important factor for controlling the resulting material size (Figure 1B). Therefore, the ratio was set to 1:160 to synthesize nanosized siRNA structures. As a result, BRCs have an overall size of 350 nm in diameter.

Optical and electron microscopes, transmission electron microscopy‐based energy dispersive X‐ray scattering (TEM‐based EDX), and dynamic light scattering (DLS) were used to characterize the BRCs in a range of aspects. Scanning electron microscopy (SEM) of the BRCs revealed their spherical structures with highly porous surface (Figure 1C). To examine the internal structures, the BRCs were observed by TEM (Figure 1D). The high magnification TEM image confirmed that the internal structure was packed uniformly. Furthermore, TEM‐based EDX analysis showed that the BRCs are composited with carbon (C), nitrogen (N), oxygen (O), phosphorous (P), and magnesium (Mg), as shown in Figure 1E. This indicates that this nanostructure is actually made up of RNA strands because N and P are the signature chemicals of the base and backbone of RNA molecules. In addition, the presence of Mg in the BRCs indicates that the structure was generated by RCT‐induced self‐assembly because the cationic ion (Mg^2+^) plays an important role in packing the RNA strands due to its negative charge.^15^ Furthermore, the surface zeta potential of the BRCs was ≈−13.2 mV, also indicating that the nanoparticles are comprised of negatively charged RNA molecules (Figure S1, Supporting Information).

In addition, atomic force microscopy (AFM), which has a good *z*‐axial resolution, was used to analyze the longitudinal dimension of the BRCs (Figure 1F). Cross‐sectional analysis of the AFM image (Figure 1G) and the overall height of the BRCs in the 3D AFM image (Figure S2, Supporting Information) showed that the overall *z*‐dimension of the BRCs was ≈350 nm, which is in accordance with the size obtained from the microscopy images, DLS analysis (Figure S3, Supporting Information), and nanoparticle tracking analysis (NTA) (Figure S4, Supporting Information). The low PDI also indicates that the RNA structures are synthesized uniformly (Figure S3, Supporting Information). Furthermore, Brownian motion of each nanoparticle was also tracked by NTA (Figure 1H; Video S1, Supporting Information), proving that the BRCs were dispersed evenly in solution.

Before evaluating its therapeutic efficacy, the in vitro siRNA generation was assessed by treating the BRCs with the Dicer enzyme. The Dicer enzyme is part of the RNase III family, and it cleaves dsRNA to short dsRNA fragments with lengths of 20–25 bp with a two‐base overhang on the 3′ end.^16^ As described in **Figure** **2**A, preprogrammed siRNA precursors within the BRCs could be processed by the Dicer enzyme. Short‐hairpin RNA‐NPs (shRNA‐NPs) were also synthesized to compare the efficiency of Dicer processing (Figure S5, Supporting Information). In the time‐course experiment, BRCs showed maximal siRNA generation at 48 h with ≈95% of the total RNA cleaved to 23 bp‐long functional siRNA molecules (Figure S6, Supporting Information). On the other hand, Dicer processing was relatively slow with the shRNA‐NPs, which results in the slow release of siRNAs from the nanoparticles (Figure 2B). Furthermore, the total functional siRNA molecules generated in 48 h were 3 times higher for the BRCs compared to shRNA‐NPs, even though the same amount of starting nanostructures had been treated with the same concentration of Dicer enzyme, indicating that the BRCs have a higher capacity to quickly release the siRNAs into cells. Melting point analysis (Figure S7, Supporting Information) and serum digestion result (Figure S8, Supporting Information) indicate that the dicer‐cleavable double‐stranded form was more stable both thermally and physiologically with the bubbled structures than hairpin structures.

**Figure 2:**
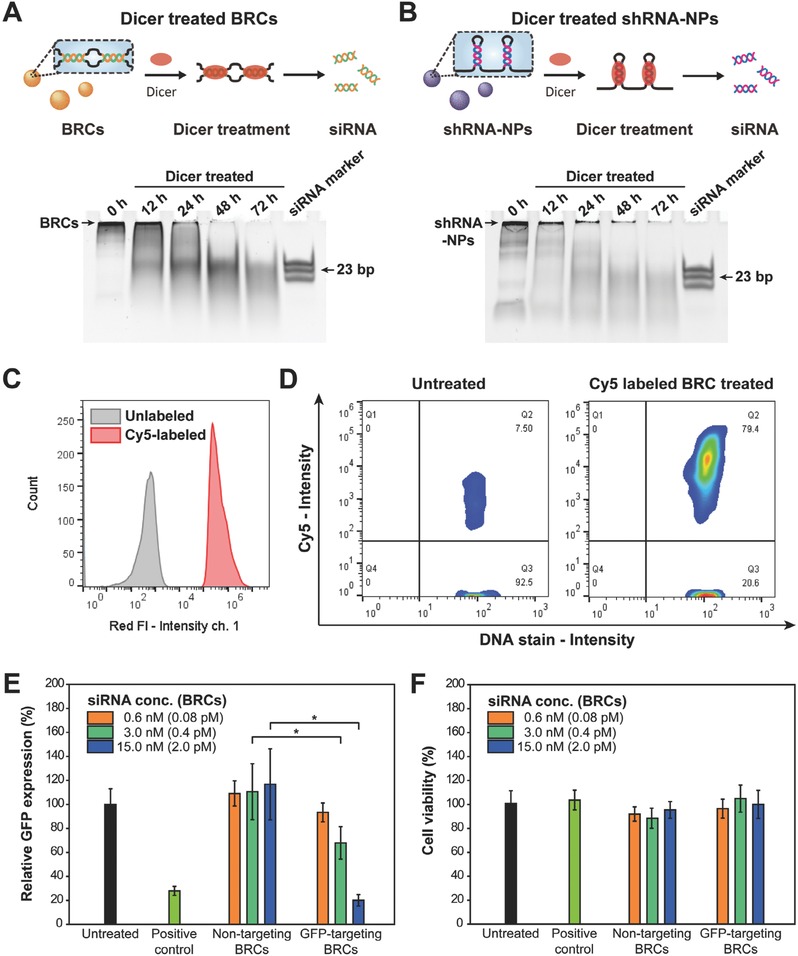
Comparison of the Dicer‐mediated in vitro generations of siRNAs from BRCs and shRNA‐NPs. A) Time‐course of siRNA generation from the BRCs and B) shRNA‐NPs, as confirmed by PAGE, along with schematic illustrations. C) Red fluorescence intensities of unlabeled (gray) and cy5‐labeled (red) BRCs, detected by cytometry. D) Cytometry analysis of HeLa cells left untreated (left) or treated with cy5‐labeled BRCs (right) for 8 h. E) Relative GFP expression (*p<0.05) and F) cell viabilities of HeLa‐GFP cells treated with GFP‐targeting or nontargeting BRCs, 50 × 10^−9^ m of siRNA monomer (positive control) or left untreated for 24 h. The same amount of siRNA is theoretically produced from BRCs at the concentration in parentheses.

To examine the cellular uptake of the BRCs covered with the transfection reagent, the BRCs were first labeled fluorescently with cyanine5 (cy5). During the RCT process, cy5‐labeled uridine triphosphate (cy5‐UTP) was added to the label, resulting in RNA molecules labeled with cy5. The fluorescently labeled BRCs were then analyzed by cytometry (Figure 2C). The increased red fluorescence intensity compared to the unlabeled BRCs indicates that the BRCs had been labeled successfully with cy5. Moreover, the involvement of cy5‐UTP in the synthesis of BRCs also indicates that the BRCs were actually comprised of the transcribed RNA strands from the RCT reaction.

Cytometry analysis (Figure 2D) and fluorescence microscopy imaging (Figure S9, Supporting Information) of the HeLa cells treated with the cy5 labeled BRCs showed that the BRCs had been internalized successfully. For the straightforward testing of the potential therapeutic efficacy of BRCs, green fluorescence protein (GFP) was set as the target gene, and the suppression of the green fluorescence intensities of the RNA nanostructure‐treated GFP‐expressing HeLa (HeLa‐GFP) cells was analyzed (Figure S10, Supporting Information). Although the BRCs with the nontargeting sequence had a negligible effect on the GFP expression level when used to treat the HeLa‐GFP cells, GFP‐targeting BRCs suppressed the GFP expression level by 80% (Figure 2E) without affecting the cell viability (Figure 2F). The gene suppression efficiency of the BRCs was slightly higher than that of the commercially available siRNA strands (positive control). The mRNA expression level was also detected by polyacrylamide gel electrophoresis (PAGE) for the quantitative analysis of the target gene knockdown effect (Figure S11, Supporting Information). When normalized to level of glyceraldehyde 3‐phosphate dehydrogenase (GAPDH) expression as a housekeeping gene, the target gene expression level was suppressed by 60%. In vitro gene suppression analysis indicated that the BRCs had high therapeutic potential without causing damage to the cells.

To examine the in vivo RNAi ability of BRCs, noninvasive real‐time fluorescence imaging was performed from the subcutaneous implantation of nude mouse models of GFP‐expressing HeLa after the single intratumoral administration of GFP‐targeting BRCs (0.8 fmole of BRCs per mouse) (**Figure** **3**A,B). The in vivo gene silencing activity of the GFP‐targeting BRCs was monitored for 4 d by measuring the GFP signal intensity in the tumor on a daily basis. The GFP signal in the GFP‐targeting BRC injected tumor was reduced gradually and diminished completely to less than 10% at 4 d after a single treatment, while the PBS or nontargeting BRC treated tumors showed no significant reduction of the GFP signal during the measurement period. This suggests that BRCs can efficiently deliver the active form of siRNA molecules to the intended cancer cell target, leading to the successful in vivo RNAi of GFP. The specific and effective silencing of GFP by the GFP‐targeting BRCs was confirmed further by both ex vivo fluorescence imaging and a histological examination of the harvested tumors (Figure 3C,D). Compared to the PBS and nontargeting BRCs, displaying bright tumor fluorescence, the GFP‐targeting BRCs exhibited the almost complete disappearance of the GFP signal from the entire tumor tissue. In addition, the quantitatively measured GFP mRNA expression pattern was consistent with the in vivo and ex vivo imaging results. As shown in Figure 3E, GFP‐targeting BRCs exhibited more than 60% GFP gene silencing, while the GFP regulation process of nontargeting BRCs remained largely unaltered, showing that the target gene silencing by BRCs occurs in a sequence‐specific manner based on RNAi. Overall, BRCs were applied successfully as an in vivo RNAi agent in the tumor xenograft model, highlighting their potential applicability as RNAi‐based therapeutics across a wide range of solid tumors.

**Figure 3:**
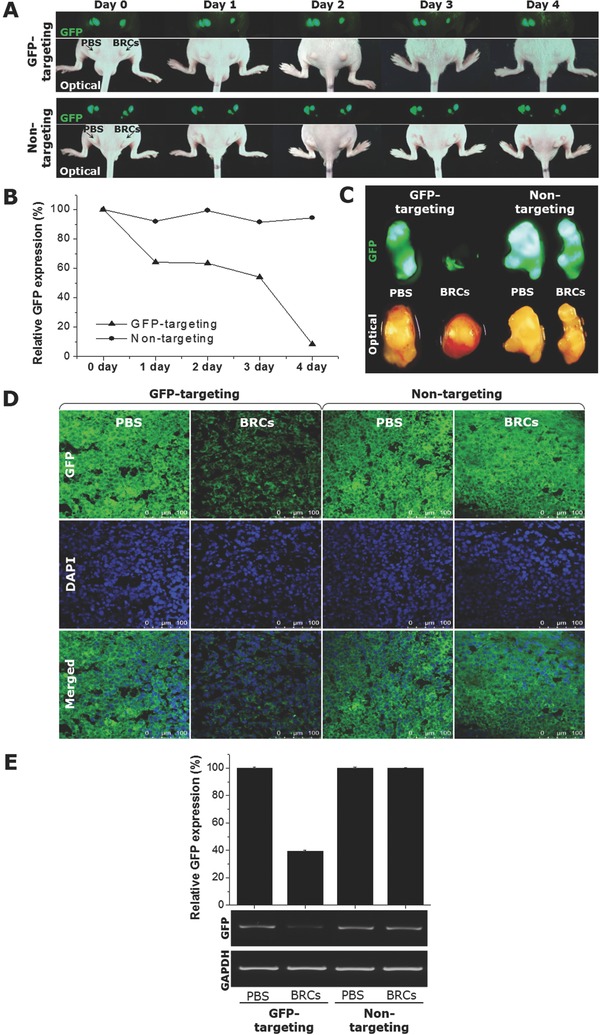
In vivo gene silencing effects of BRCs in nude mice bearing GFP‐HeLa xenograft tumors. A) Real‐time in vivo imaging of GFP‐HeLa tumors treated with BRCs containing GFP‐targeted or nontargeted siRNAs. The mice were analyzed at the indicated times after an intratumoral injection of phosphate‐buffered saline (PBS) and 0.8 fmole of BRCs. B) Relative GFP signal intensities at the tumor tissue, plotted as the percent change in GFP‐targeting versus nontargeting as a function of time. C) Ex vivo imaging of GFP‐HeLa tumors excised on day 4 postinjection. D) Confocal microscopy of the GFP‐HeLa tumor sections from the excised tumor tissues 4 d post‐treatment. The tumor tissues were stained with 4′,6‐diamidino‐2‐phenylindole (DAPI) solution for locating cell nucleus (blue). Original magnification, ×400. E) Reverse transcription polymerase chain reaction (RT‐PCR) analysis of the GFP mRNA expression levels in GFP‐HeLa tumors injected with PBS and BRCs. The band intensity representing GFP was normalized to that of GAPDH, which was used as an internal standard, and is expressed as the percentage change relative to the controls (PBS).

In summary, bubbled RNA‐based cargoes were synthesized by an enzymatic approach with a preprogrammed sequence for the specific cleavage sites for Dicer to process. The overall size of the nanoparticles was controlled rationally by adjusting the ratio of template DNAs to the T7 RNA polymerase in the RCT reaction. The BRCs were synthesized by a one‐pot process, which avoided the labor‐intensive synthetic process, as is the case for many synthetic processes of other types of nanoparticles. Moreover, the resulting nanoparticles have a narrow size distribution, and the efficient Dicer‐mediated release of siRNAs from the BRCs was confirmed in vitro. The gene‐specific knockdown was also confirmed in vivo by an analysis of the gene silencing activity of GFP‐targeting BRCs, compared to the nontargeting BRCs. Overall, this study showed that the BRCs can be synthesized and purified easily, and this system can be utilized for target‐specific RNAi therapeutics with high efficiency in the generation of functional siRNAs in the target cells.

## Experimental Section

The methods detailing the BRC synthesis, characterization, Dicer‐mediated siRNA generation, and in vitro and in vivo gene knockdown analysis are included in the Supporting Information.

## Supporting information

Supplementary

Supplementary
