# Supplementary material for: Bubbled RNA‐Based Cargo for Boosting RNA Interference
Source: Adv Sci (Weinh). 2017 Mar 27;4(8):1600523. doi: 10.1002/advs.201600523 (PMC5566230; doi:10.1002/advs.201600523)
Supplement: Supplementary file 1 — Supplementary [file ADVS-4-na-s001.pdf]

Copyright WILEY-VCH Verlag GmbH & Co. KGaA, 69469 Weinheim, Germany, 2017.

ADVANCED SCIENCE
Open Access

Supporting Information

for *Adv. Sci.,* DOI: 10.1002/advs.201600523

Bubbled RNA-Based Cargo for Boosting RNA Interference

Hyejin Kim, Jaepil Jeong, Dajeong Kim, Gijung Kwak, Sun Hwa Kim, and Jong Bum Lee**

Supporting Information

Bubbled RNA-based Cargo for Boosting RNA Interference

Hyejin Kim[1], Jaepil Jeong[1], Dajeong Kim[1], Gijung Kwak[2, 3], Sun Hwa Kim[3,], Jong Bum Lee[1,*]*

[1] Department of Chemical Engineering, University of Seoul, 163 Seoulsiripdaero, Dongdaemun-gu, Seoul 02504, Republic of Korea

[2] KU-KIST Graduate School of Converging Science and Technology, Korea University, 145 Anam-ro, Seongbuk-gu, Seoul 02841, Republic of Korea

[3] Center for Theragnosis, Biomedical Research Institute, Korea Institute of Science and Technology (KIST), Seoul 02792, Republic of Korea

*Correspondence and requests for materials should be addressed to J.B.L. (e-mail: jblee@uos.ac.kr) and S.H.K. (e-mail: sunkim@kist.re.kr).

Experimental Section

Sequence design for BRCs

Template linear DNAs are designed to have four different regions that are complementary to the following RNA sequence: 1) T7 promoter, 2) sense or anti-sense strand of siRNAs, 3) bubble (non-complementary) and 4) sense or anti-sense strand of siRNAs. All DNA sequences are listed in Table S1. In result, RNA strands synthesized by T7 RNA polymerase have the following sequence: [siRNA-bubble-siRNA-bubble]$_n$.

Synthesis of BRCs

For the synthesis of BRCs, linear DNA1 and linear DNA2 are circularized as previously reported [1]. Then, circular DNA1 and circular DNA2 at the final concentrations of 0.5 μM were mixed with 8 mM of ribonucleotide solution mix (New England BioLabs), reaction buffer (80 mM Tris, 40 mM NaCl, 12 mM $MgCl_2$, 4 mM spermidine, 20 mM dithiothreitol, pH 7.8) and 80 units μl^{-1} of T7 RNA polymerase (Ambion). For the RCT reaction, the reaction solution was incubated for 20 h at 37°C. The final reaction solution was briefly sonicated, then the BRCs were washed three times with nuclease-free water before further analysis. For the synthesis of cy5-labeled BRCs, cy5-UTP (final concentration of 20 μM) was added to the RCT reaction mixture at the beginning of the incubation process. To remove unincorporated cy5-UTP, the BRCs were washed three times with nuclease-free water after RCT reaction.

Characterization

FE-SEM (Hitachi, S-5000H) and AFM (Park Systems, Park NX10) were used to obtain high resolution digital images of the BRCs. The BRCs for SEM imaging were deposited onto silicon wafer, and coated with Pt after being dried. The nanoparticles for AFM imaging were deposited

onto freshly cleaved mica (Ted Pella), and air-dried. The samples were scanned in non-contact mode with NC-NCH tips (Park Systems). Nanoparticle tracking analysis (NTA) was carried out with NanoSight NS300 (Malvern). TEM (JEOL, JEM-2100F) was employed to characterize the BRCs operating at an accelerated voltage of 200 kV. For the preparation of samples, the BRCs were deposited onto a Lacey Formvar/carbon-coated copper grids (Ted Pella, 01883-F), then air-dried at room temperature.

Dicer-mediated in vitro generation of siRNAs

For assessment of siRNA generation from BRCs, the nanoparticles were incubated with 0.1 U μl^{-1} of recombinant human Dicer enzyme and reaction buffer (1 mM ATP, 2.5 mM $MgCl_2$, 40% Dicer reaction buffer) at 37ºC for 24 h. The cleaved RNAs were examined by 10% non-denaturing PAGE in 0.5X Tris-borate-EDTA (TBE) buffer carried out at 120 V for 60 min. Then, the gels were stained with 1X GelRed in 0.5X TBE and analyzed under UV with GelDoc EZ Imager (Bio-Rad). The lane profiles were analyzed by GelDoc software (Bio-Rad).

Calculation of amount of siRNA in BRCs

RNA contents in BRCs were measured with UV-Vis spectrophotometer (NanoDrop 2000c, Thermo Fisher Scientific) by measuring absorbance at 260 nm, and BRC particle concentration was measured by NTA. From the measured RNA contents and BRC particle concentration, RNA contents in a single BRC can be calculated as follows:

$$\text{RNA contents in a single BRC } \left({}^{g\text{ RNA}}\!/_{\text{BRC particle}}\right)$$

$$= \text{RNA contents } \left({}^{g\text{ RNA}}\!/_{l}\right) \times \frac{1}{\text{BRC particle concentration}} \left({}^{l}\!/_{\text{BRC particles}}\right)$$

$$= 0.21 \times 10^{-14}\ {}^{g\text{ RNA}}\!/_{\text{BRC particle}}$$

By design, a half of total RNA contents can potentially be siRNAs. The amount of siRNAs in one BRC can be calculated as follows:

$$\text{The amount of siRNAs in one BRC}$$
$$= \text{RNA contents in a single BRC} \left({}^{g\ \text{RNA}}\!/_{\text{BRC particle}} \right) \times \frac{46\ bp}{92\ bp} \times \frac{1}{M.W.of\ siRNA} \times N_A$$

Therefore, about 49000 of siRNA can be maximally generated from one BRC in theory. Experimentally, the amount of cleaved siRNA from one BRC was determined by PAGE (Figure 2A). According to the results following Dicer treatment, about 7600 siRNA strands were generated from single BRC under optimal conditions. As previously reported, some portion of the RNA is not as readily accessed by Dicer in a more close-packed self-assembled RNA structure, and multimers of repeated RNA unit as incomplete dicing products could be produced [2].

In vitro gene knockdown analysis

HeLa-GFP cells were grown in DMEM supplemented with 10% FBS, 100 U ml^{-1} of penicillin, 100 μg ml^{-1} of streptomycin and 1% Antibiotic-Antimycotic at 37ºC in a humidified atmosphere supplemented with 5% CO_2. The cells were passaged routinely to maintain exponential growth. One day prior to transfection (~90% confluence), the cells were trypsinized, diluted with fresh medium and transferred to 96-well plates (7000 cells per well). The BRCs were covered with the transfection reagent, Stemfect™ RNA Transfection Kit (Stemgent), prior to transfection according to the manufacturer's instruction. Then, the cells were treated with different concentrations, ranging from 0.08 pM to 2.0 pM (siRNA concentrations ranging from 0.6 nM to 15.0 nM) of the covered BRCs. Silencer GFP siRNA (AM4626, Ambion) was treated at the concentration of 50 nM, according to manufacturer's

recommendation, as positive control group. After 24 h of treatment, cells were washed with DPBS (14190-144, Gibco), and lysed with CelLyticM (C2978, Sigma-Aldrich). The green fluorescence from each well containing the lysed cells was detected by microplate reader (Synergy HT, BioTek), then normalized with the green fluorescence from the well containing untreated HeLa-GFP cell lysates to obtain relative GFP expression. Cell viabilities were assessed with Cell Counting Kit-8 (CK04-11, Dojindo) according to the manufacturer's instruction. To assess relative GFP/GAPDH mRNA expression level, the cells were washed and lysed, and their total RNA was extracted using a NucleoSpin RNA Plus (740984.10, Macherey-Nagel) after 24 h of the treatment with anti-GFP BRCs. RNA content was measured using a UV-Vis spectrophotometer (NanoDrop 2000c, Thermo Fisher Scientific), and cDNA was synthesized from equal amounts of RNA using High Capacity RNA-to-cDNA kit (4387406, Applied Biosystems) according to the manufacturer's protocol. The cDNAs were amplified by Taq DNA polymerase (S26030, Intron Biotechnology), and the assay was normalized by amplifying the housekeeping gene (GAPDH). For observing GFP knockdown in HeLa-GFP cells, the cells were seeded at 8-well chamber slide at the density of 40000 cells per well. The cells were treated with 2.0 pM of the covered BRCs for 24 h. The cells were then stained with Hoechst33342, and observed with fluorescence microscopy (Eclipse Ti-U, Nikon).

In vivo gene knockdown analysis

The in vivo gene silencing effects of BRCs were evaluated using GFP-HeLa tumor-bearing mouse model. GFP-expressing HeLa cells (1×10^6 cells/flank) were inoculated to both flanks of six-week-old Balb/c nude mice. A week after the day of the first GFP fluorescence detection in tumor, mice were injected intratumorally by PBS or 0.8 fmole of BRCs covered with the same transfection reagent used for in vitro assays. Then the GFP/HeLa-bearing mice were scanned using a non-invasive whole-body animal imaging system (eXplore Optix system) on

days 0, 1, 2, 3 and 4. Total photon flux in GFP-HeLa tumors was quantified by analyzing regions of interest using Analysis Workstation software (ART Advanced Research Technologies Inc., Montreal, Canada). A quantification of in vivo GFP gene silencing in GFP-HeLa xenograft tumors was recorded as total photons per centimeter squared per steradian (p/s/cm^2/sr) per each tumor. Four days post-injection, mice were sacrificed and tumor tissues were extracted and further examined for ex vivo tissue imaging. Fluorescence signal in the excised tumor tissues was monitored using a 12-bit CCD camera (Image Station 4000 MM; Kodak, New Haven, CT). Half of each of the tumor tissue was fixed in 10% buffered formalin and paraffin-embedded using standard procedures for confocal microscopic analysis and the other half of the tumor was used for quantitative RT-PCR assays. For microscopic image analysis of tumor tissues, the sections (6 mm) were cut from each block and stained with DAPI (4′,6-diamidino-2-phenylindole) solution. The GFP expression in tumor tissues was visualized by a confocal laser scanning microscope (Olympus Fluoview FV300, Melville, NY) using an argon/krypton mixed gas laser (ex. 488 nm). To examine GFP mRNA expression levels with quantitative RT-PCR analysis, total RNA was isolated from tumor tissues using the RNeasy® Mini Kit (Qiagen, Valencia, CA), according to the manufacturer's recommendation. One microgram of the isolated RNA was reverse transcribed using SuperScript™ III (Invitrogen, Carlsbad, CA) and random hexamers, according to the manufacturer's protocol. PCR was performed (Invitrogen, Carlsbad, CA) on 2 ml of cDNA using Platinum Taq Polymerase (Invitrogen). PCR amplification was performed under the following thermal cycling conditions: Taq activation; 95ºC for 5 min, PCR amplification; 20 cycles for GAPDH, 25 cycles for GFP at 95ºC for 1 min, at 60ºC for 1 min, and at 72ºC for 2 min, final extension; 1 cycle 72ºC for 5 min. The PCR products were separated in a 1.0% agarose gel by electrophoresis and visualized with ethidium bromide staining.

Table

Table S1. DNA sequences for synthesizing BRCs and shRNA-NPs. Complementary DNA sequence for promoter region for T7 RNA polymerase is shown in red, and primer for T7 RNA polymerase binds to the red region to form promoter region for T7 RNA polymerase. Complementary DNA sequence for sense and anti-sense strands for siRNAs are presented as purple and orange, respectively.

**DNA strands**	**Length (nt)**	**Sequence**
Linear DNA 1 for GFP-targeting BRCs (sense)	92	5'- Phosphate - ATA GTG AGT CGT ATT AAA AAC TTC AGG GTC AGC TTG CTT GCT GGA TGA AGG ACG GTC GAA CGC AAA ACT TCA GGG TCA GCT TGC TTA TCC CT - 3'
Linear DNA 2 for GFP targeting BRCs (anti-sense)	92	5'- Phosphate - ATA GTG AGT CGT ATT AAA GCA AGC TGA CCC TGA AGT TTT CTT AGG CTG GAC AAC AAC CAT CTA AAG CAA GCT GAC CCT GAA GTT TTA TCC CT - 3'
Linear DNA 1 for non-targeting BRCs (sense)	92	5'- Phosphate - ATA GTG AGT CGT ATT AAA ATG TGA ATG CAG ACC AAA GAA TTG CTG GAT GAA GGA CGG TCG AAA ATG TGA ATG CAG ACC AAA GAA TTA TCC CT - 3'
Linear DNA 2 for non-targeting BRCs (anti-sense)	92	5'- Phosphate - ATA GTG AGT CGT ATT AAA TTC TTT GGT CTG CAT TCA CAT TTG GCT GGA CAA CAA CCA TCT AAA TTC TTT GGT CTG CAT TCA CAT TTA TCC CT - 3'
Linear DNA for GFP-targeting shRNA-NP	92	5'- Phosphate - ATA GTG AGT CGT ATT AAC GTA CCA ACA AAA CTT CAG GGT CAG CTT GCT TAC TTG AAG CAA GCT GAC CCT GAA CTT TTT AGA GGC ATA TCC CT - 3'
Primer for T7 RNA polymerase	22	5' - TAA TAC GAC TCA CTA TAG GGA T - 3'
GAPDH forward primer	20	5' - ACC ACA GTC CAT GCC ATC AC - 3'
GAPDH backward primer	20	5' - TCC ACC ACC CTG TTG CTG TA - 3'
GFP forward primer	20	5' - AAG TCG TGC TGC TTC ATG TG - 3'
GFP reverse primer	20	5' - ACG TAA ACG GCC ACA AGT TC - 3'

Figures

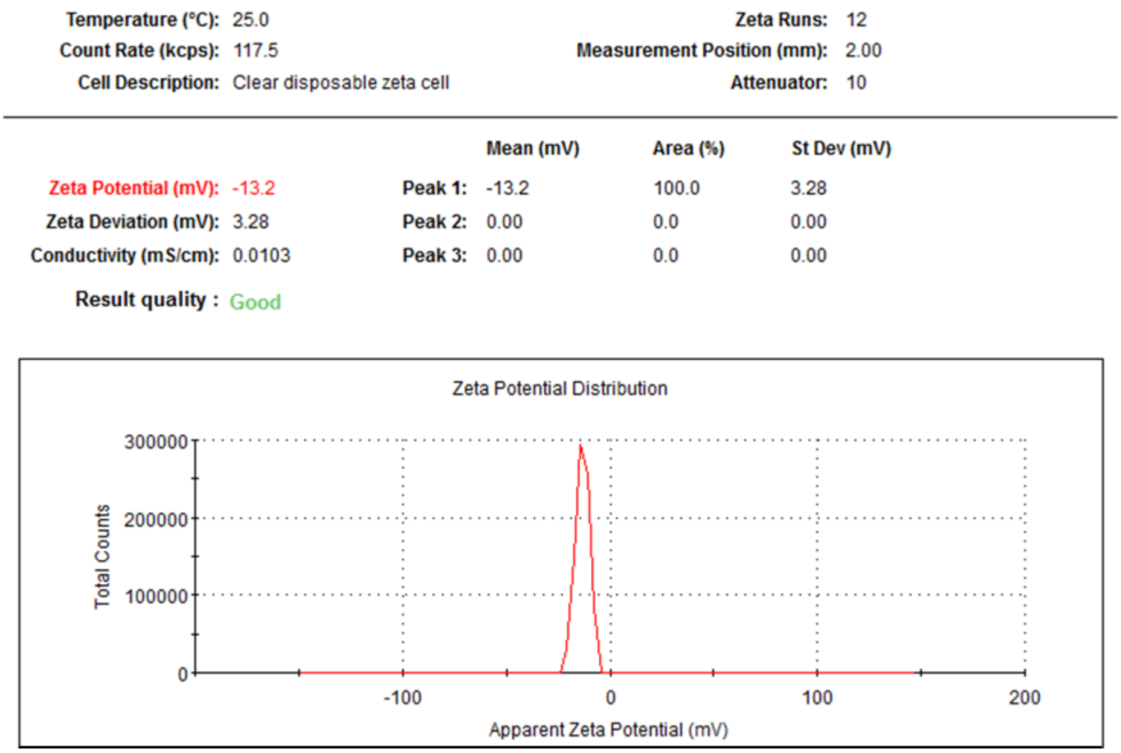

Figure S1. Zeta potential analysis showing that the average zeta potential of BRCs is -13.2 ± 3.28 mV. The zeta potential has negative value due to phosphate backbones of the packed RNA strands comprising BRCs.

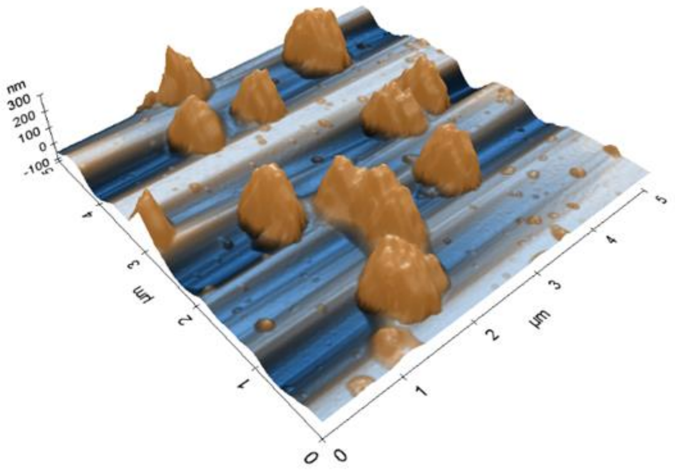

Figure S2. AFM image shown in 3D revealing that the overall height of the BRCs is about 350 nm.

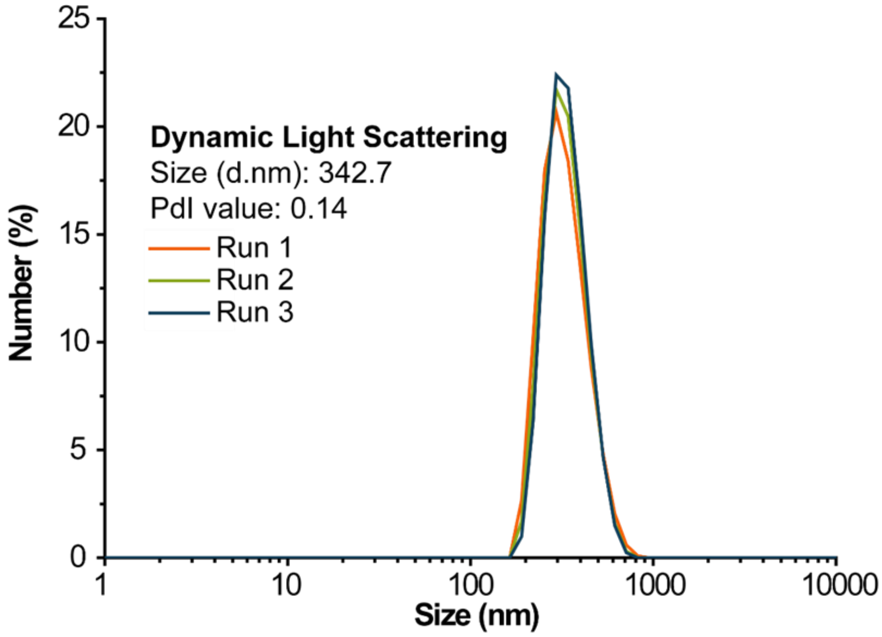

Figure S3. Dynamic light scattering analysis revealing the size distribution of BRCs. The average size of the BRCs was 342.7 nm and polydispersity index (PdI) value was 0.14, indicating that the BRCs have narrow size distribution.

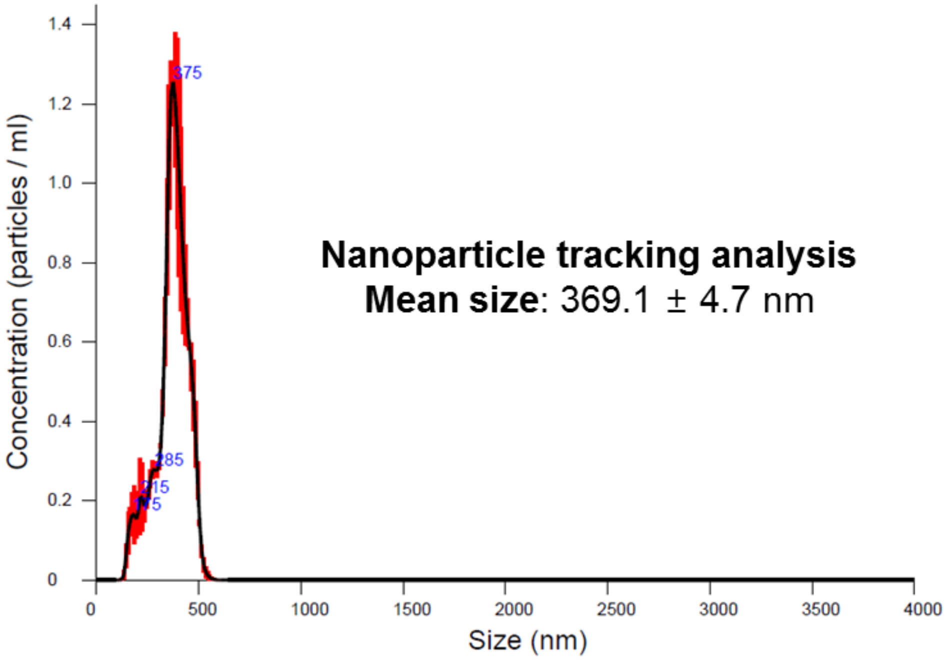

Figure S4. Nanoparticle tracking analysis showing that the average size of BRCs are 369.1 ± 4.7 nm.

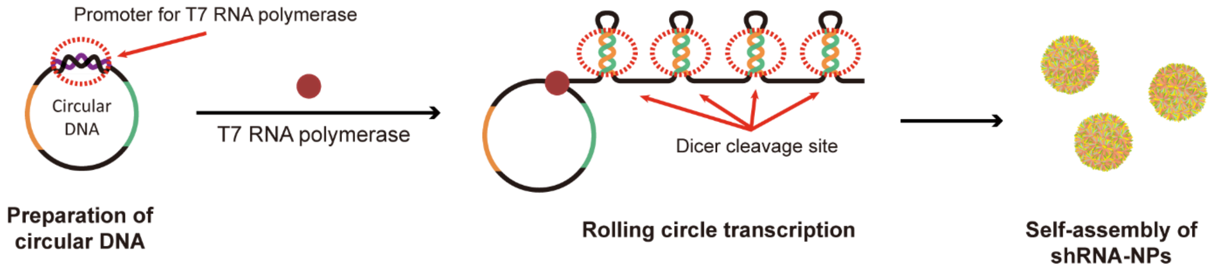

Figure S5. Schematic illustration of synthesizing short-hairpin RNA nanoparticles (shRNA-NPs) by RCT-induced self-assembly. Short-hairpin regions are specific cleavage sites for Dicer to generate functional siRNAs.

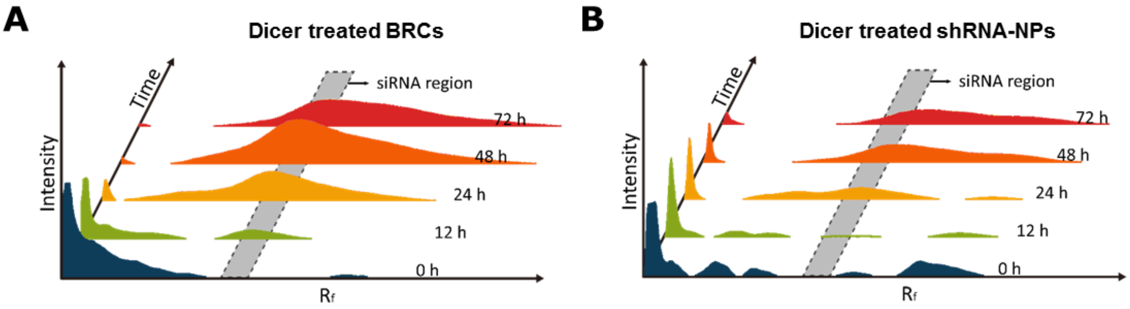

C

(band %)

Time	BRCs	shRNA-NPs
0	0.2	0.1
12	23.0	4.2
24	81.1	42.3
48	95.1	61.5
72	94.4	72.6

Figure S6. Time-dependent siRNA generation from BRCs and shRNA-NPs. (A, B) Lane profiles from PAGE analysis of in vitro siRNA generation from BRCs (A) and shRNA-NPs (B), revealing relative amount of siRNA generation from BRCs and shRNA-NPs. (C) Percentages of the bands at the location of 23 bp in PAGE analysis (Figure 2A, 2B) of BRCs and shRNA-NPs treated with Dicer enzyme, indicating 95.1% of total RNA has been processed to have a length of 23 bp.

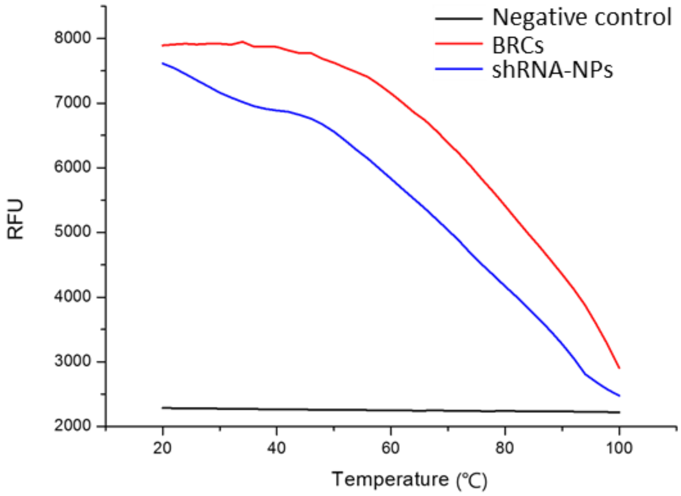

Figure S7. Melting point analysis of the BRCs and shRNA-NPs. The value of relative fluorescence unit (RFU) is stable upto 40ºC or BRCs, while the value of RFU is decreasing at all temperatures for shRNA-NPs. This result indicates that the double-strand formation is more favorably occurred in the synthetic process of BRCs than that of shRNA-NPs, though the same mass of siRNA precursors are intended to form in both cases.

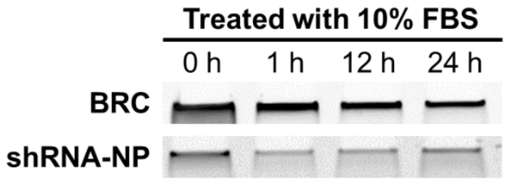

Figure S8. Stability of BRCs and shRNA-NPs under physiological condition. 2 μg of BRCs and shRNA-NPs were incubated with 10% FBS at 37˚C for 0 h, 1 h, 12 h or 24 h. To cease serum nuclease-medicated digestion of the nanostructures, RNase inhibitor (1 unit per μl) was added after the reaction. And the resulting solution was analyzed with 10% non-denaturing PAGE, and the band intensities were analyzed by GelDoc Software. After 24 h of 10% FBS treatment, 93% of BRCs and 46% of shRNA-NPs were remained intact.

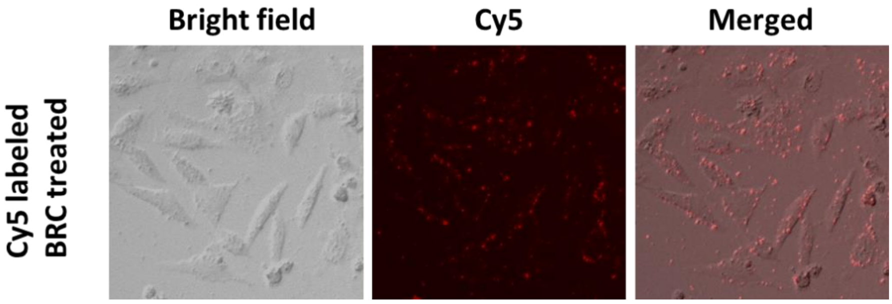

Figure S9. Fluorescence microscopy images of HeLa cells treated with cy5-labeled BRCs for 8 h. Cells have cy5 signals within cytoplasm, but not in the region of nucleus, indicating the BRCs are localized successfully in cytoplasm.

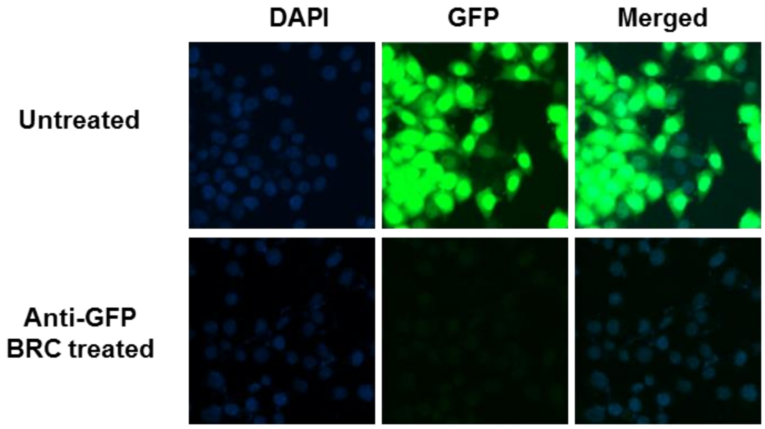

Figure S10. Fluorescence microscopy images of HeLa-GFP cells treated with anti-GFP BRCs or left untreated for 24 h.

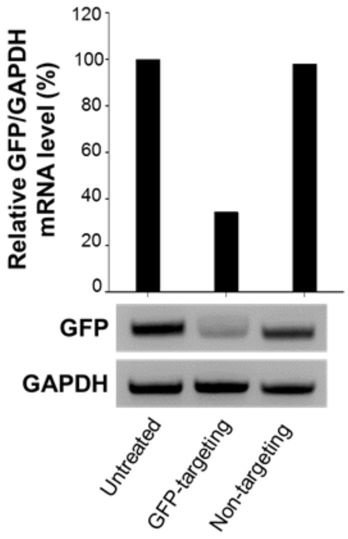

Figure S11. Relative GFP mRNA expression level of HeLa-GFP cells treated with GFP-targeting or non-targeting BRCs for 24 h, normalized with GAPDH mRNA expression level.

References

1. Lee, J.B., et al., A mechanical metamaterial made from a DNA hydrogel. Nat. Nanotechnol. 7, 816-820 (2012).

2. Lee, J.B., et al., Self-assembled RNA interference microsponges for efficient siRNA delivery. Nat. Mater. 11, 316-322 (2012).
